# A word to the wise …

**DOI:** 10.1093/icvts/ivac252

**Published:** 2022-10-07

**Authors:** Augusto D’Onofrio, Gino Gerosa

**Affiliations:** Division of Cardiac Surgery, University of Padova, Padova, Italy; Division of Cardiac Surgery, University of Padova, Padova, Italy

**Keywords:** Mitral valve repair, Microinvasive cardiac surgery

We read with interest Dr. David’s commentary [1] about our recently published paper entitled ‘Transapical beating heart mitral valve repair versus conventional surgery: a propensity-matched study’ [2]. We would like to clarify some aspects that in our opinion are crucial.

We are afraid that Dr. David missed one of the key points of our study that, despite its intrinsic limitations that limit the generalizability of our results, shows that in patients with favourable anatomy (prolapse of the P2 segment), there seem to be no differences in terms of freedom from recurrent mitral regurgitation and of freedom from reoperation. Therefore, despite the worse outcomes in the overall population (this is what Dr. David refers to in his commentary), in well-selected patients, transapical beating heart mitral valve repair with neochords implantation (NC) provides similar outcomes to conventional surgery up to 5 years.

We respectfully disagree with Dr. David when he states that NC should be selected in inoperable patients only after excluding the feasibility of a transcatheter Mitraclip (Abbott laboratories, Chicago, IL, USA). There are no data supporting this statement since there are no studies comparing NC and Mitraclip. However, in a recently published article about 100 NC patients with 5-year follow-up (including technical and patient-selection learning curves), we found an incidence of reoperation and of severe mitral regurgitation (MR), in patients with favourable anatomy, of 14.7% and 14.7%, respectively [3]. On the other hand, in the EVEREST-II study (therefore in highly selected patients), rates of reoperation and 3+ or 4+ MR at 5 years in the percutaneous repair group were 43% and 19%, respectively [4]. Noteworthy, 3+ and 4+ MR do not include moderate MR but only moderate–severe and severe MR and also, the EVEREST-II included both degenerative and functional MR. Another important aspect to consider is that so far over 100,000 patients over a 17-year period have been treated with Mitraclip, demonstrating the perseverance of our interventional cardiology colleagues (and some surgeons) that, despite the poor initial outcomes of this procedure, have worked to transform a suboptimal conventional surgical procedure (edge to edge with no annular stabilization) into a successful microinvasive alternative, optimizing technology, technique and patient selection [5, 6]. Meanwhile, NC procedures have been performed only in ∼1000 patients and we are still using the first-generation device. There are still many aspects that may be improved in order to achieve better results.

We certainly agree that conventional surgery so far provides unsurpassed optimal outcomes for degenerative MR, especially if performed in high-volume centres and by highly committed surgeons or, even better, by world-recognized Masters as Dr. David. His results are impressive but the real world is a different thing and such numbers are difficult to replicate. The truth is that not all tennis players are Novak Djokovic or Rafa Nadal and not all basketball players are Lebron James or Michael Jordan. Clearly, experience and case load play a fundamental role in all surgical procedures, conventional, minimally invasive and microinvasive but, in our opinion, surgeons should not remain stuck in their positions pretending that conventional cardiac surgery is always the only and best solution. There are now several microinvasive possibilities for many diseases and the ability to correct structural heart defects on the beating heart and off-pump is now a reliable option. How many surgeons really believed in transcatheter aortic valve replacement 15 years ago? Let us be honest, very few and this is why we are now struggling. Percutaneous transseptal mitral NC has already been performed in humans … just a word to the wise ….

## Data Availability

No new data were generated or analysed in support of this research.
